# Supplementary material for: Alleviation Syndrome of High-Cholesterol-Diet-Induced Hypercholesterolemia in Mice by Intervention with Lactiplantibacillus plantarum WLPL21 via Regulation of Cholesterol Metabolism and Transportation as Well as Gut Microbiota
Source: Nutrients. 2023 Jun 1;15(11):2600. doi: 10.3390/nu15112600 (PMC10255518; doi:10.3390/nu15112600)
Supplement: Supplementary file 1 [file nutrients-15-02600-s001.zip › nutrients-2407452-supplementary.pdf]

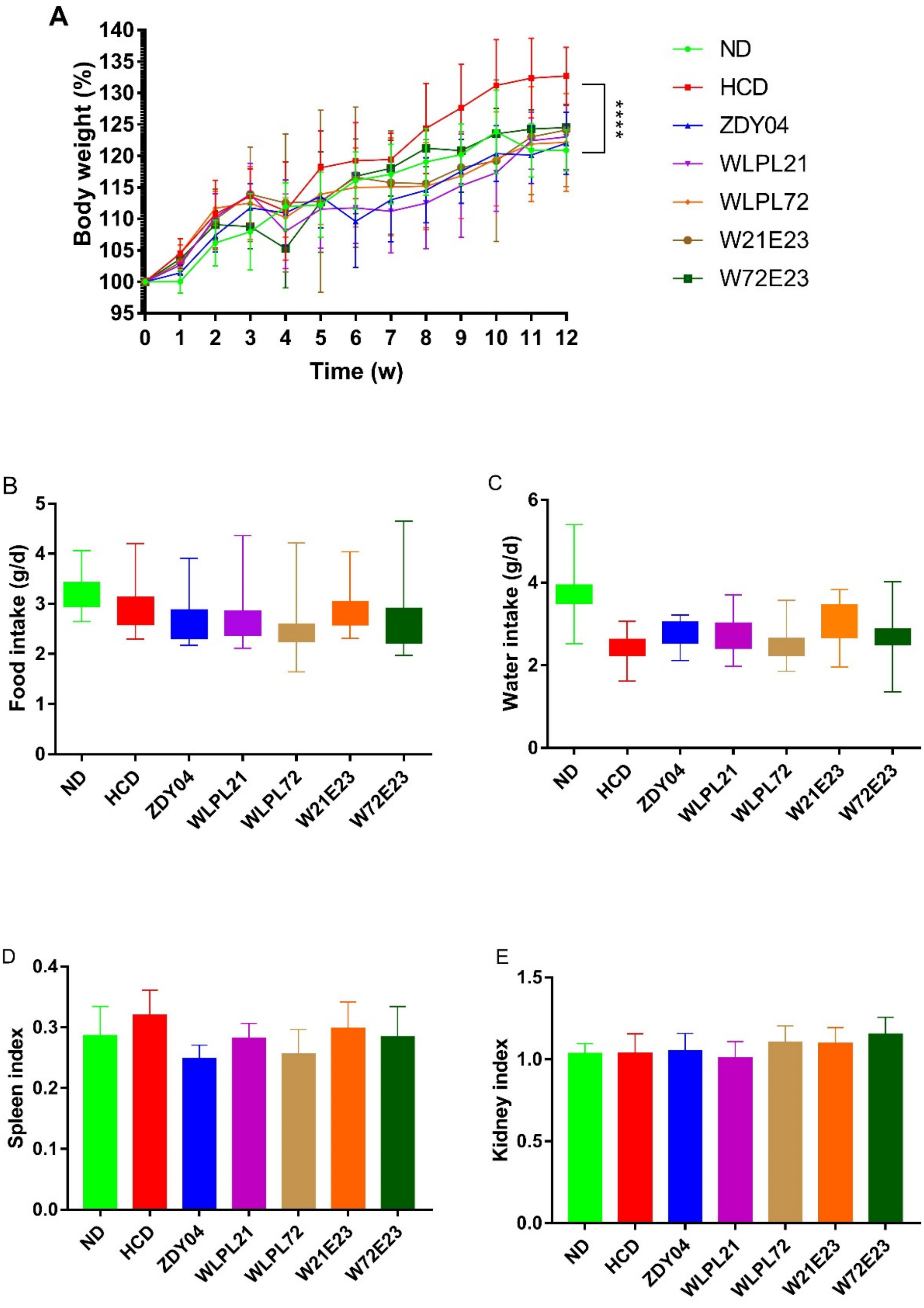

Figure S1. Effect of probiotics on physics and visceral index in high cholesterol diet-induced hypercholesterolemia. Body weight (A), food intake (B) and water intake were recording. Index of spleen (D) and kidney(E) was indicated as ratio of organ/weight × 100. ND: normal diet; HCD: high cholesterol diet; ZDY04: HCD with *Lactiplantibacillus plantarum* ZDY04; WLPL21: *L. plantarum* WLPL21; WLPL72: *L. plantarum* WLPL72; W21E23: HCD with bacterial cocktail of *L. plantarum* WLPL21 and *Enterococcus faecium* WEFA23; W72E23: HCD with bacterial cocktail of *L. plantarum* WLPL21 and *E. faecium* WEFA23. Data were expressed as mean ± SD. ****$p < 0.0001$, data in HCD were compared to HCD.

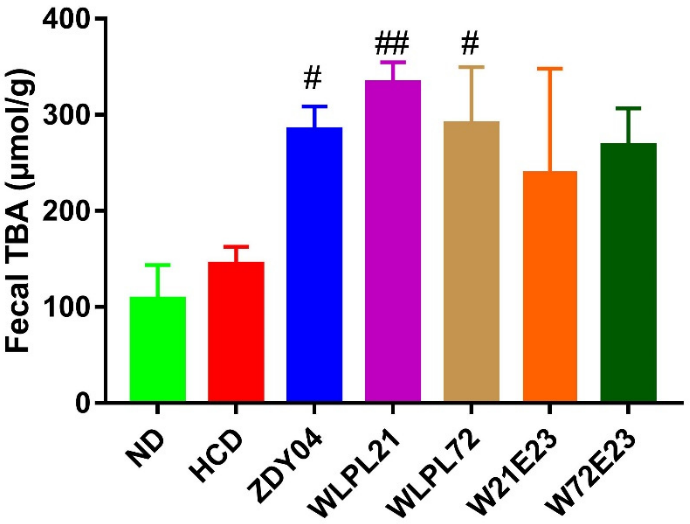

Figure S2. Effect of probiotics on cholesterol metabolism by detecting total bile acid (TBA) in fecal. ND: normal diet; HCD: high cholesterol diet; ZDY04: HCD with *L. plantarum* ZDY04; WLPL21: *L. plantarum* WLPL21; WLPL72: *L. plantarum* WLPL72; W21E23: HCD with bacterial cocktail of *L. plantarum* WLPL21 and *E. faecium* WEFA23; W72E23: HCD with bacterial cocktail of *L. plantarum* WLPL21 and *E. faecium* WEFA23. Data were expressed as mean ± SD. $^{\#}p < 0.05$, $^{\#\#}p < 0.01$, data in probiotic groups were compared to HCD.

A

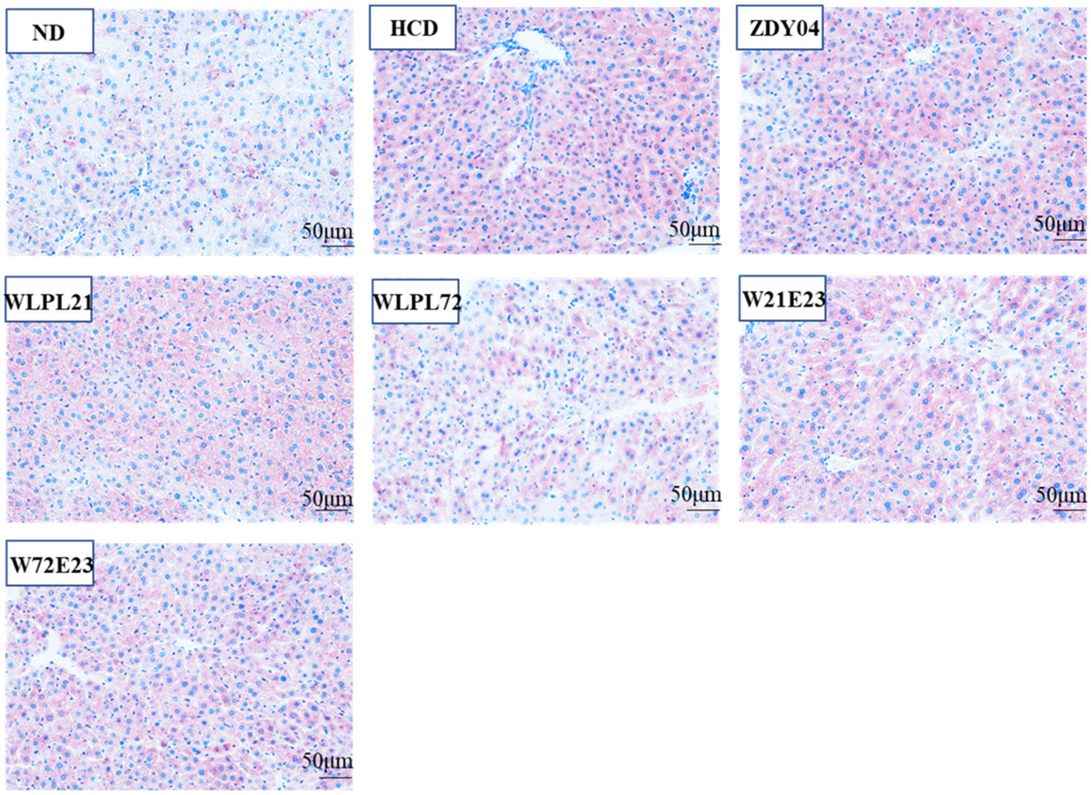

B

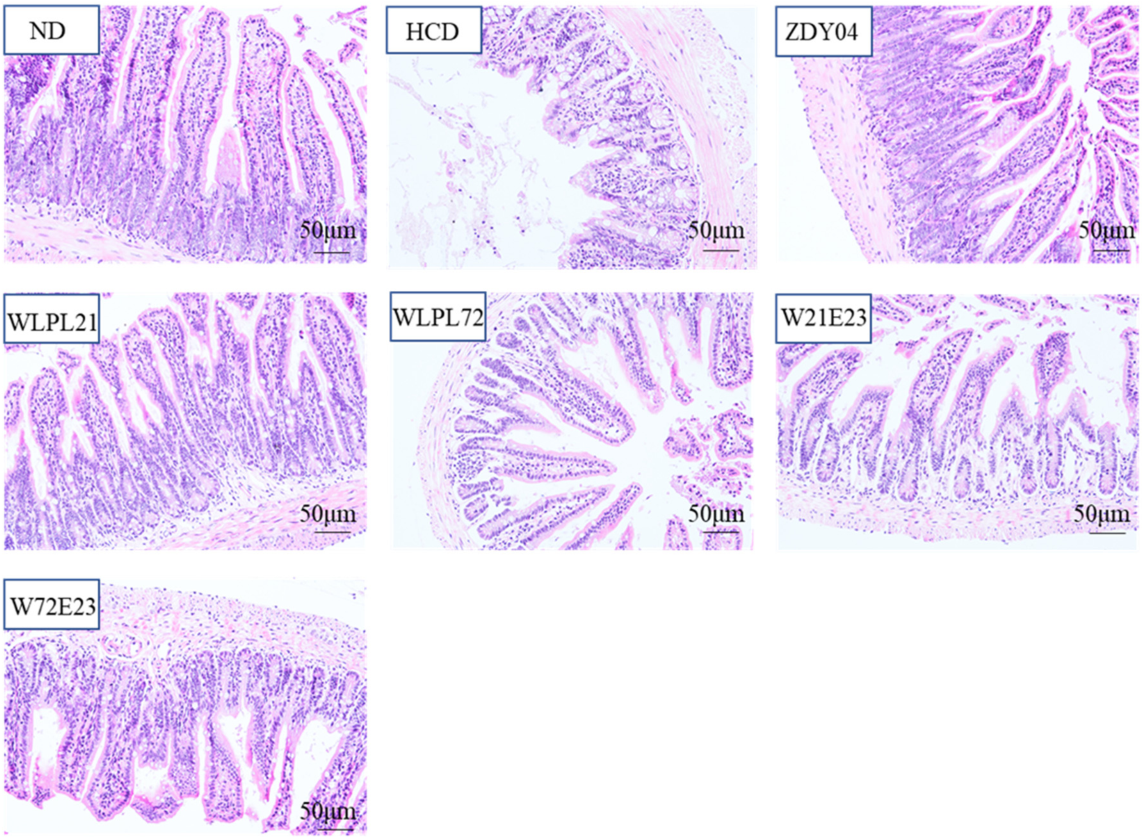

Figure S3. Effect of probiotics on hepatic lipid deposition detected by oil Red O (A) (× 200) and histology of intestine tissue analyzed by H & E (B) (× 200) in hypercholesterolemic mice. ND: normal diet; HCD: high cholesterol diet; ZDY04: HCD with *L. plantarum* ZDY04; WLPL21: *L. plantarum* WLPL21; WLPL72: *L. plantarum* WLPL72; W21E23: HCD with bacterial cocktail of *L. plantarum* WLPL21 and *E. faecium* WEFA23; W72E23: HCD with bacterial cocktail of *L. plantarum* WLPL21 and *E. faecium* WEFA23.
